# Markers of T-cell senescence and physical frailty: insights from Singapore Longitudinal Ageing Studies

**DOI:** 10.1038/npjamd.2015.5

**Published:** 2015-09-28

**Authors:** Tze Pin Ng, Xavier Camous, Ma Shwe Zin Nyunt, Anusha Vasudev, Crystal Tze Ying Tan, Liang Feng, Tamas Fulop, Keng Bee Yap, Anis Larbi

**Affiliations:** 1 Gerontology Research Programme, Department of Psychological Medicine, National University Health System, Yong Loo Lin School of Medicine, National University of Singapore, Singapore, Singapore; 2 Singapore Immunology Network, Aging and Immunity Program, Agency for Science Technology and Research, Singapore, Singapore; 3 Research Center on Aging, Faculty of Medicine and Health Sciences, University of Sherbrooke, Quebec, Canada; 4 Department of Geriatric Medicine, Alexandra Hospital, Ministry of Health, Singapore, Singapore

## Abstract

**Background:**

Elderly individuals have an eroded immune system but whether immune senescence is implicated with the development of frailty is unknown. The underlying immune mechanisms and the link between markers of senescence and physical frailty is not well established.

**Methods:**

We explored the association of specific T-cell subset markers of immune differentiation and senescence on CD4^+^ and CD8^+^ cells (CD28^−^, CD27^−^ and CD57^+^) and the immune risk profile (inverted CD4/CD8 ratio <1) with physical frailty among 421 participants who were frail (*N*=32), prefrail (*N*=187) and robust (*N*=202) in the Singapore Longitudinal Ageing Study cohort.

**Results:**

In ordinal logistic regression models relating tertile category rank scores of immune biomarker with frailty status (robust, prefrail and frail), CD8^+^CD28^−^CD27^+^ (odds ratio (OR)=1.35, *P*=0.013), CD4^+^CD28^−^CD27^+^ (OR=1.29, *P*=0.025), CD8^+^CD28^−^ (OR=1.31, *P*=0.022), and CD4/CD8 ratio (OR=1.27, *P*=0.026) were positively associated with frailty, controlling for age, sex and multimorbidity. CD4/CD8 ratio less than one was not associated with frailty (OR=0.84, *P*=0.64). In stepwise multinomial logistic regression controlling for age, sex and comorbidity, only CD8^+^CD28^−^CD27^+^ was the independent predictor of prefrailty: highest tertile of the immune marker significantly predicted prefrailty (versus low tertile, OR=1.72, *P*=0.037) and frailty (OR=2.56, *P*=0.06).

**Conclusion:**

The study supports the hypothetical role of immune senescence in physical frailty, particularly in regard to the observed loss of CD28 expression from both CD8^+^ cells and CD4^+^ cells, but not for CD27 or CD4/CD8 ratio as a marker of senescence. The potential of CD8^+^CD28^−^CD27^+^ as a biological marker of frailty should be further investigated in prospective studies.

## Introduction

Frailty is a geriatric syndrome which may be described as a nonspecific state of increased vulnerability resulting from decreased physiological reserves, multisystem dysregulation and limited capacity to maintain homeostasis.^1,2^ Frail older persons are vulnerable to increased risk of hospitalization, dependency, institutionalization and deaths when exposed to stress. There is strong evidence to suggest that chronic inflammation, indicated by levels of interleukin-6 (IL-6) and C-reactive protein (CRP) and increased white blood cell counts, is a key pathophysiological factor in the frailty syndrome.^3,4^ However, the underlying immune mechanisms that contribute to frailty and the pathways to chronic inflammation in older adults are still not well understood.

The age-associated loss of immunity known as immune senescence is strongly associated with higher vulnerability for severe infections,^5^ vaccine failure^6^ and premature mortality^7^ in older individuals. Clinically, these individuals are often considered as frail and at-risk for diseases. Immunosenescence is marked by a progressive decrease in the number of naive cells and an increase in the number of memory cells showing poor functionality. The differentiation of T cells toward a senescent phenotype is characterized typically by clonal expansion of CD8^+^ T lymphocytes that lack expression of CD28.^8,9^ CD28 is an important co-stimulatory molecule for T-cell activation, and a marker of the proliferative history of cells. Low expression levels of the CD27 are also regarded as a marker of senescence.^10–12^ CD27 is another co-stimulatory molecule and tumor necrosis factor (TNF) receptor required for generation and long-term maintenance of T-cell immunity by regulating B-cell activation and immunoglobulin synthesis.

Increased CD8^+^CD28^−^ is strongly associated with seropositivity to persistent infections such as cytomegalovirus (CMV),^13–15^ suggesting that CMV infection may be a driving force behind the accumulation of the CD8^+^CD28^−^ effector cell expansions.^16–18^ The loss of the CD28 marker is also shown to be associated with increased CD57 expression on T cells^13,19^ which is a marker for replicative senescence. In older persons, CMV serological positivity is associated with significant increases in the number of CD8^+^CD57^+^CD28^−^ T cells.^20^ A reduced CD4/CD8 ratio and increased CD8^+^CD28^−^ lymphocytes are regarded as key markers of the immune risk profile.^14,16^ Although low CD4/CD8 ratio is associated with increased mortality^13,15^ the CD8^+^CD28^−^ lymphocyte subset is also well characterized particularly for its associations with vaccine failure.^21^ The status of the CD4^+^CD27^−^ T-cell subset as a clinical marker of immune risk profile and the CD4^+^ population in general is less well characterized in the field of aging.

So far, the link between biological parameters of immunosenescence and the physical frailty syndrome is not well proven by the small number of published studies. Low total lymphocyte count, a crude indicator of immune status, was inconsistently found to be associated with increased risk of frailty.^4,22,23^ In a case–control study of community-living women aged 65 years and over in the Women’s Health and Aging Studies (WHAS), secondary exploratory analyses suggested that CD8^+^ lymphocytes and CD8^+^CD28^−^ lymphocyte counts were significantly higher among women who were frail compared with prefrail and non-frail women.^23^ However, the study was primarily designed to investigate the association of specific T-cell subsets with increased mortality, not frailty. Lower CD4 and high CD8 T-cell counts among frail older persons than matched controls were reported as an incidental finding in a small study (*n*=26) that reported their primary finding of an association between higher proinflammatory CC chemokine receptor five expressing cells and frailty.^22^ The large population Newcastle 85+ Study found limited evidence to support the role of immunosenescence among very old persons. No relationships were found with parameters of the immune risk profile (e.g., CD4/CD8<1.0) and the inverse relationships observed with memory/naive CD8 T- and B-cell ratios were in the opposite direction to that expected.^24^ To date, it is uncertain whether CD8^+^ and CD4^+^ cell subsets and the immune risk profile could be used as markers or associated with frailty.

The aim of this paper was to explore the association of specific CD8^+^ and CD4^+^ T-cell subset markers of immune senescence (CD28^−^, CD27^−^ and CD57^+^) and the immune risk profile with physical frailty, defined by a widely used clinical research definition of the frailty syndrome, based on measures of weight loss, weakness, slowness, exhaustion and reduced physical activity.^25^

## Materials and methods

### Study participants

The study was conducted using blood samples that were collected from 421 participants in the second wave recruitment cohort of the Singapore Longitudinal Ageing Studies (SLAS-2). Previous publications have detailed the SLAS study design, population sampling and measurements.^26^ In brief, all residents aged 55 years and above were identified from door-to-door census and eligible persons, excluding those with severe physical or mental disability who could not participate in the extensive questionnaire interviews or physical and neurocognitive performance tests, were invited to participate in the research. The research was approved by the National University of Singapore Institutional Review Board, and informed consent was obtained from all participants. The SLAS-2 cohort completed baseline survey for residents in the South West and South Central regions of Singapore in 2010–2013 (*N*=2010 as of 30 April 2013, response rate 78%) and used identical methodologies used in the first wave SLAS cohort (SLAS-1). The participants in this study represented a sub-sample of all residents in two precincts (Telok Blangah and Brickworks) who were recruited during the period from May 2011 to Dec 2013, and provided blood samples for this study, excluding subjects with a history of hospitalization in the past 6 months and high CRP levels (>3 mg/l). At baseline, all participants underwent five to six detailed interview sessions in their homes, and on-site clinical assessments, performance-based testing and venesection by trained research personnel for an extensive range of demographic, medical, biological, psychosocial, behavioral and neurocognitive variables.

### Measurements

#### Frailty

The physical frailty phenotype was defined using five criteria proposed and validated in the Cardiovascular Health Study (CHS):^25^ unintentional shrinking; slowness; weakness; exhaustion and low activity. The measurements used in this study to define the frailty construct were similar but not identical to those used in the original CHS study. A participant without any of the five components was defined as robust, one to two components as prefrail; three and more components as frail.

1. Unintentional shrinking: body mass index (BMI) of <18.5 kg/m^2^ and/or unintentional weight loss ⩾10 pounds (4.5 kg) in the last 6 months.
2. Slowness was assessed using 6-m fast gait speed test using the average of two measurements, and the lowest quintile values stratified for gender and height to classify participants as slow, based on data in a previous large population-based study.^27^
3. Weakness: leg muscle strength was determined using dominant knee extension, using the average value from three trials in kilograms, standardized on gender and BMI strata. Participants with knee extension strengths in the lowest quintiles were classified as weak.
4. Exhaustion was measured with three questions on vitality domain in the Medical Outcomes Study SF-12: ‘Did you feel worn out?’, ‘Did you feel tired?’, ‘Did you have a lot of energy?’ with total summed scores ranging from 3 to 15, higher score indicating more energy. A score of <10 was used to denote exhaustion.
5. Low activity: physical activities were assessed based on self-reported time (in h) spent doing light (office work, driving a car, strolling, standing with little motion, personal care and so on), moderate and vigorous activities (gardening, brisk walking, dancing, jogging, swimming, strenuous sports and so on) on weekdays and weekend. The total amount of time spent on performing moderate and vigorous activities per week and activity time below the gender-specific lowest quintile was used to denote frailty on this criterion, based on data in a previous large population-based study.^27^

#### Comorbidities

The number of medical comorbidities was determined from self-report of a history of medical conditions diagnosed by a doctor and corroborated by the use of specific medications. The principal list of major chronic diseases includes hypertension, diabetes, dyslipidemia, myocardial infarction/ heart failure, stroke, cancer, asthma /chronic obstructive lung disease kidney failure, hip fracture, arthritis and other medical diagnoses specified by the participants. Over-weight or obese was defined using body mass index ⩾25 kg/m2. Multi-comorbidity was defined as 2 or more comorbid conditions (versus one or no medical conditions).

### Sample preparation

Blood from overnight fasting participants was drawn in CPT tubes (BD Biosciences), and were transported and processed within 2 h, according to manufacturer’s instructions. After centrifugation at 1,650 r.p.m. for 20 min at room temperature, plasma and peripheral blood mononuclear cells (PBMCs) were collected. Plasma was stored in −80 °C, whereas PBMCs were washed twice in PBS and cryopreserved in liquid nitrogen. Freezing was performed by keeping cells in 90% fetal bovine serum (FBS) containing 10% dimethyl sulfoxide. On the day of experiments, cryovials were thawed rapidly and extensively washed with PBS containing 10% FBS. Samples usually offer a recovery higher than 75% with no specific loss of immune population.^28^ Viability was higher than 95% as tested by trypan blue exclusion.

### CRP measurement

Frozen plasma samples were thawed and diluted 1:100 in appropriate buffer. CRP was measured by Luminex in 1:5 dilute plasma with appropriate standards as per manufacturer’s recommendation (Biorad, USA).

### Flow cytometry staining

PBMCs were counted and allowed to rest for 2 h in FACS buffer (PBS containing 10% FCS, 5 mM EDTA and 2 mM azide). For each staining 1×10^6^ PBMCs were used. The staining includes the following markers: CD3-PECy5.5 (Beckman Coulter, USA), CD4-PECy7 (Biolegend, USA), CD8-APCCy7 (BD Biosciences, USA), CD45RA-eFluor605 (eBiosciences, USA), CD57-Pacific Blue (Biolegend), CD28^−^PETexasRed (Beckman Coulter), CD27^−^APC (Biolegend). All staining performed included a Live/Dead marker (Invitrogen, USA) to exclude false positive staining. The CD4/CD8 ratio was measured in whole blood to match with previous studies.^16^ Briefly, 100 μl whole blood were stained with CD3-PECy5.5, CD4-PECy7 and CD8-APCCy7 for 20 min at 4 degrees followed by incubation with lysis buffer (eBiosciences) to eliminate red blood cells. Flow Cytometry data were analyzed using FlowJo (Treestar, USA), FACSDiva (BD Biosciences) and Kaluza (Beckman Coulter) (Supplementary Figure S1).

### Data analysis

Exploratory data analyses included the determination of the skewed distributions for some T-cell subset variables and took into account the ordinal values of the frailty scores, and bivariate analysis of zero-order and age-adjusted partial correlations of T-cell subset immune markers with frailty score, with statistical significance assessed by Holm–Bonferroni correction for multiplicity. Nominally significant T-cell subsets were selected as candidate variables for further examination in multiple ordinal and multinomial logistic regression analyses with ordered categories of frailty (0, 1–2, 3) as dependent variable, adjusting for age, sex and multimorbidity. The independent variables were analyzed as tertile rank scores (1, 2, 3) and ordinal tertile categories (low, mid and high tertiles) of T-cell immune markers. Odds ratio (OR) of association with 95% confidence intervals (95% CIs) of the relationships between individual T-cell subset variables with frailty, adjusted for age, sex and multimorbidity.

## Results

The study participants (52% females, mean age of 66.5 years) included *n*=32 (7.6%) frail, *n*=187 (44.4%) prefrail and *n*=202 (48%) robust older persons (Supplementary Table S1). As expected, frail and prefrail participants were significantly older and had more medical comorbidities (Supplementary Table S2). The frail individuals were significantly older (mean age 74.5 years, *P*<0.0001). While only 10% of the robust participants displayed multi-comorbidity, nearly one third of the frail participants have multi-comorbidity (*P*<0.004). The range of specific medical morbidities among the robust, prefrail and frail groups were shown in Supplementary Table S3. Females were equally distributed in the different groups.

In exploratory zero-order and age-adjusted partial correlational analyses, (Table 1) significant associations at nominal *P*<0.05 with frailty score were found for the frequency of CD8^+^CD28^−^CD27^+^ and CD4^+^CD28^−^CD27^+^ T cells, and for the CD4/CD8 ratio. The relationships with age of these T-cell subsets are also shown in the Supplementary Figure S2. Marginally, CD8^+^CD28^−^ was nominally associated at *P*=0.017 in zero-order correlation and *P*=0.065 in age-adjusted partial correlation. CD4^+^ T cells tend to lose CD27 expression while CD8^+^ T cells will first lose CD28 expression during differentiation. However, the CD4^+^CD27^−^ population we tested did not show correlation with frailty. There was no correlation between one of the most accepted marker for immunosenescence in T cells, CD57, and frailty. Detailed examination of their correlations with frailty components (BMI, gait velocity, energy, physical activity and knee extension strength) and their dependence with age, sex and medical morbidities are presented in Table 2. Significant correlations with frailty components were observed particularly for knee extension strength (all T-cell subsets including CD8^+^CD28^−^) and physical activity score (CD8^+^CD28−CD27^+^). CD8^+^CD28^−^CD27^+^ was significantly associated with overall frailty score (*P*=0.003). Also, there was a significant but unexpectedly positive association of CD4/CD8 ratio with frailty score (*P*=0.004). Overall, frailty and knee extension strength are highly correlated to T-cell populations lacking CD28 expression.

Table 3 presents the results of ordinal logistic regression for ORs of association of ordinal categorical levels of CD4/CD8 ratio, CD8^+^CD28^−^CD27^+^, CD4^+^CD28^−^CD27^+^ and CD8^+^CD28^−^T cells with ordinal categories of frailty status (robust, prefrail and frail). Sex, age and multimorbidity were included as fixed covariates in the base model. These revealed that tertile levels of CD4^+^CD28^−^CD27^+^ (OR=1.29, *P*=0.025) and CD8^+^CD28^−^CD27^+^ (OR=1.35, *P*=0.013), and CD8^+^CD28^−^ (OR=1.31, *P*=0.022) were significantly associated with frailty status, independently of sex, age and multimorbidity. CD4/CD8 ratio, against expectation was also significantly but positively associated with frailty (OR=1.27, *P*=0.026). Finally, multinomial logistic regression models (Table 4) were used to determine the strongest independent T-cell marker(s) predicting prefrailty or frailty. Sex, age and number of comorbidities analyzed as fixed variables in the base model, and both forward conditional and backward conditional stepwise selections of immune markers (tertiles of CD4/CD8 ratio, CD4^+^CD28^−^CD27^+^, CD8^+^CD28^−^CD27^+^ and CD8^+^CD28−) with *P*<0.05 for entry and 0.10 for retention were used. The frequency of CD8^+^CD28^−^CD27^+^ cells was the only independent significant predictor of prefrailty and frailty: high tertile of the immune marker significantly predicted prefrailty versus low tertile, OR=1.72 (*P*=0.037) and better predicted frailty with OR=2.56 (*P*=0.06).

## Discussion

The data in this study support the hypothetical role of immune senescence in physical frailty, particularly in regard to the observed loss of CD28 expression from CD8^+^ cells as well as CD4^+^ cells.^13,14^ Both subsets of CD8^+^CD28^−^CD27^+^ and CD4^+^CD28^−^CD27^+^ consistently showed a positive association with frailty. The consistent trend of association of CD8^+^CD28^−^ cell counts with increasing frailty corroborates the results from the Women Health and Ageing Study.^23^ The negative relationship between CD8^+^CD28^+^CD27^+^ and frailty, although not statistically significant, is also consistent with the notion that the preservation of a large population of naive and early memory cells (central memory) is associated with delaying the development of frailty in old age. The clonal expansions of CD28^+^CD57^+^ cells and CD8^+^CD28^−^ effector cells are both closely associated with CMV infection,^8,9^ strongly suspected to be a cause of lifelong latent infection and immune erosion in late life. In this study, there was no prima facie association between CD8^+^CD57^+^ T cells and frailty. CD57 is a marker of replicative senescence and usually highly expressed in elderly individuals or patients with persistent infections such as CMV. The lack of association for CD57 in our study suggests that the events/mechanisms leading to nonphysiological aging (frailty) and events leading to accelerated biological aging (immunosenescence), although occurring in parallel, are independent.

We found no strong support for an association of CD27 alone with frailty in this study. Hitherto, high expression levels of the CD27 combined with low levels of KLRG1 expression are regarded as hallmarks for fit memory cells,^11^ and senescent memory cells that have lost proliferative potential have been reported to express low amounts of CD27 and high levels of KLRG1.^10–12^ Recent studies, however, show that CD27 and KLRG1 expression patterns at the memory stage merely reflect the initial priming conditions of a T-cell when exposed to inflammation, and do not accurately predict T-cell protective and proliferative potential.^29^ Because they do not reflect functional properties and are not dictated by division history as currently postulated, it has been suggested that currently used biomarkers of senescence based on CD27 (and KLRG1) expression patterns should not be used to predict memory development or fitness.^29^ This appears to be borne out by the results of this study.

To our knowledge, this is the first study to identify CD8^+^28^−^ T-cell immune markers of senescence as predictors of frailty. A key pathophysiological factor of the physical frailty syndrome is systemic inflammation characterized by increased levels of proinflammatory cytokines, such as TNF-alpha (TNF-a) and other inflammatory molecules which increase the production of interleukin-6 (IL-6) and CRP, all of which have catabolic effects on muscles.^30^ Increased levels of proinflammatory cytokines may also be the result of cellular senescence associated with ageing, which although beneficial for tumor suppression, leads to the acquisition of a senescent-associated secretory phenotype, and a striking increase in the secretion of proinflammatory cytokines.^31^ It has been shown that TNF-a induces a significant quantitative reduction of CD28 molecules on the cell surface that leads to the emergence of CD28^−^ CD8 T cells.^32^ CD8^+^CD28^−^ T cells may therefore be generated in proinflammatory environment associated with ageing and frailty.

Downregulation of CD28 in CD8^+^ T cells is also associated with reduced telomere length and altered telomerase activity.^33^ Short telomeres and increased telomerase activity was shown to be the hallmark of increased allostatic load and poor resilience.^34^ In a very large study it was shown that telomere length in whole blood was not associated with frailty.^35^ However, the study did not measure telomere length in subpopulations of circulating cells but in whole blood. Blood is composed of a variety of immune cells including a majority of neutrophils that are short lived cells in which telomere length is not affected by the aging process. In addition, the previous studies may use different approach to classify frailty. On the basis of our study, the measure of immune aging using surface markers such as CD28 provided a better association to clinical outcomes such as frailty than more complex interactive markers such as telomere/telomerase.

Recently, murine studies have reported the increased expression of programmed cell death-1 (PD-1) receptor specifically on exhausted CD8^+^ effector memory T cells with low levels of CD28 showing limited proliferative and cytokine-producing capacity.^36^ As the increased expression of PD-1 is likely due to chronic activation of the immune system from lifelong latent infection associated with ageing and frailty, further work on the identification of a CD8^+^CD28^−^ coupled with PD1^+^ positivity as a replicative senescence phenotype in frailty may be fruitful.

Contrary to expectation, we found that CD4/CD8 cell ratio was positively associated with frailty, controlling for age, sex and multimorbidity. Furthermore, CD4/CD8 cell ratio less than one (a critical parameter of the immune risk profile) was not associated with increased odds of association with frailty. This result is in agreement with the large population Newcastle 85+ Study which also found a similar positive trend of association for increasing CD4/CD8 ratio with frailty (although not statistically significant), and no relationships between CD4/CD8 ratio less than one and frailty.^24^ However, secondary analyses of data of women aged 65 and over in the WHAS did observe that CD4/CD8 ratio was inversely associated with increasing frailty, measured using the same CHS criteria.^23^ An inverted CD4/CD8 ratio is regarded as a key immune senescence marker as it was predictive of higher mortality in the Swedish OCTO and NONA longitudinal studies of people aged over 85.^14,15^ However, the WHAS found no significant differences in CD4/CD8 ratio and other T-cell subsets to explain early mortality among its cohort members who were followed up over 5–7 years. Further studies are needed to establish whether the CD4/CD8 ratio as a predictive marker distinguishes risks of frailty and mortality.

Several aspects of the study design and population should be noted in interpreting and evaluating the results in this study. The study was based on a large population-based sample hence the findings are widely generalizable across a wide range of frailty status. The mean age of the study participants (66 years) is relatively young, and the varying contribution of immune senescence to frailty development across the age range is unclear. For example, whether the IRP applies in an age group before 80 years old is still controversial and most studies use poor outcomes such as mortality and few have shown a predictive value of the IRP at the presymptomatic level. Participants who were unable to participate in the study due to severe physical and/or mental disability due to terminal stage chronic illnesses such as cancer and dementia were likely to include frail individuals with cachexia, hence the observed relationships were likely be biased towards the null finding. Because of the cross-sectional design of the study, these initial findings will need to be confirmed in larger prospective studies.

We found that CD8^+^CD28^−^CD27^+^ was a specific T-cell subset that was strongly predictive of frailty, independent of sex, age and multimorbidity. This population represents a population of T cells with a strong replicative history but it is unclear why these cells expand in frail individuals. Whether they have limited specificity and may originate from persistent pathogens is a possibility. Akbar *et al.* have shown that retaining CD27 expression in CD28^−^ T cells was associated with sustained Akt activity. Knowing the role of the Akt pathway in regulating glycolysis and mTOR, a metabolic relationship between immune senescence and frailty is possible. Further studies should be conducted to establish this potential ‘immune frailty’ as a prognostic indicator of adverse health outcomes of frailty.

## Figures and Tables

**Table 1:** Correlations of T-cell subsets with frailty score (0–5)

	*Zero order*		*Controlling for age*	
	*Spearman’s r*	P-*value*	*Partial* r	P-*value*
%CD4	0.112	0.021	0.075	0.124
%CD8	−0.112	0.021	−0.075	0.124
CD4/CD8 ratio	0.112	0.021	0.146	0.003^a^
CD8^+^CD28^+^CD27^−^	−0.081	0.097	−0.062	0.201
CD8^+^CD28^+^CD27^+^	−0.095	0.051	−0.037	0.452
CD8^+^CD28^−^CD27−	0.047	0.334	0.014	0.779
CD8^+^CD28^−^CD27^+^	0.128	0.008^a^	0.153	0.002^a^
CD8^+^CD28^−^	0.116	0.017	0.090	0.065
CD8^+^CD57^+^	0.088	0.070	0.016	0.737
CD4^+^CD28^+^CD27^−^	−0.042	0.386	−0.062	0.201
CD4^+^CD28^+^CD27^+^	−0.040	0.415	0.017	0.721
CD4^+^CD28^−^CD27^−^	0.005	0.921	−0.008	0.865
CD4^+^CD28^−^CD27^+^	0.098	0.044	0.097	0.047
CD4^+^CD27^−^	−0.015	0.764	−0.055	0.257
CD4^+^CD57^+^	0.004	0.939	−0.046	0.343

a Statistically significant by Holm–Bonferronni corrected *P-*value <0.0042.

**Table 2:** Detailed analyses of candidate immune markers: correlations with clinical risk factors and physical frailty components

	*Clinical risk factors* *Frailty components*	*CD4/CD8 ratio*		*CD4* *^+^* *CD28* ^−^ *CD27* *^+^*		*CD8* ^+^ *CD28* *^−^* *CD27* *^+^*		*CD8* *^+^* *CD28* *^−^*	
		*Rho*	P-*value*	*Rho*	P-*value*	*Rho*	P-*value*	*Rho*	P-*value*
Zero order correlations	Female sex	0.038	0.430	0.002	0.970	0.020	0.680	0.005	0.92
	Age	−0.065	0.181	−0.055	0.260	0.037	0.440	0.167	0.001
	No. of medical morbidities	−0.025	0.610	−0.102	0.037	−0.018	0.710	0.063	0.194
	Frailty score	0.112	0.021	0.098	0.044	0.128	0.008	0.116	0.017
	BMI	0.122	0.012	−0.036	0.457	−0.029	0.560	−0.096	0.05
	6-m fast gait, s	0.113	0.021	0.054	0.270	0.110	0.024	0.061	0.215
	Energy	−0.004	0.930	0.002	0.960	−0.032	0.520	−0.057	0.244
	Physical activity score	0.076	0.121	0.025	0.610	−0.062	0.204	−0.051	0.297
	Knee extension strength	−0.061	0.213	−0.112	0.021	−0.141	0.004	−0.208	0.001
Partial correlations controlling for age, sex and number of comorbidities	Frailty score	0.139	0.004	0.094	0.055	0.147	0.003	0.086	0.080
	BMI	0.145	0.003	−0.035	0.474	−0.056	0.250	−0.091	0.060
	6-m fast gait, s	0.147	0.003	0.026	0.590	0.092	0.060	0.043	0.390
	Energy	−0.008	0.870	0.015	0.760	−0.048	0.330	−0.054	0.270
	Physical activity score	0.056	0.250	−0.002	0.960	−0.116	0.018	−0.042	0.390
	Knee extension strength	−0.120	0.014	−0.129	0.008	−0.156	0.001	−0.148	0.002

Abbreviation: BMI, body mass index.

**Table 3:** Ordinal logistic regression odds ratio of association of T-cell subsets with frailty status (robust, prefrail and frail)

*T-cell subset markers*	*Unadjusted*			*Adjusted for age, sex and multimorbidity*		
	*OR*	*95% CI*	P-*value*	*OR*	*95% CI*	P-*value*
CD4/CD8 ratio (tertile score: 1,2,3)	1.18	0.96–1.45	0.108	1.27	1.03–1.57	0.026
Low tertile	1			1		
Mid tertile	1.06	0.61–1.84	0.84	1.09	0.62–1.93	0.75
High tertile	1.41	0.93–2.12	0.102	1.63	1.07–2.48	0.023
CD4/CD8<1.0 (binary: 0,1)	0.71	0.36–1.42	0.33	0.84	0.42–1.71	0.64
CD4^+^CD28−CD27^+^ (tertile scores: 1,2,3)	1.25	1.00–1.55	0.049	1.29	1.03–1.62	0.025
Low tertile	1			1		
Mid tertile	1.45	0.91–2.29	0.115	1.65	1.03–2.64	0.037
High tertile	1.55	1.00–2.41	0.052	1.66	1.06–2.61	0.027
CD8^+^CD28−CD27^+^ (tertile scores:1,2,3)	1.35	1.07–1.70	0.012	1.35	1.06–1.71	0.013
Low tertile	1			1		
Mid tertile	0.80	0.50–1.26	0.33	0.84	0.53–1.34	0.47
High tertile	1.81	1.14–2.88	0.012	1.80	1.13–2.89	0.014
CD8^+^CD28− (tertile scores: 1,2,3)	1.42	1.13–1.78	0.002	1.31	1.04–1.66	0.022
Low tertile	1			1		
Mid tertile	1.44	0.91–2.30	0.123	1.28	0.80–2.06	0.30
High tertile	2.01	1.28–3.17	0.002	1.72	1.08–2.74	0.022

Abbreviations: CI, confidence interval; OR, odds ratio.

Ordinal logistic regression models were fitted with Frailty status (0, 1–2, 3–5) as the ordinal dependent variable and tertile categories of immune biomarker as the independent variable was used as a ranked score independent variable. Age, sex and number of comorbidities were included as fixed covariates in the models.

**Table 4:** Multinomial logistic regression analyses of significant T-cell marker of Prefrailty and Frailty from stepwise forward selection

	*Prefrailty*				*Frailty*			
	*OR*	*95%*	*CI*	P-*value*	*OR*	*95%*	*CI*	P-*value*
Male sex	1.01	0.67	−1.55	0.94	0.56	0.24	−1.32	0.184
Age, years	1.01	0.98	−1.04	0.52	1.16	1.10	−1.22	0.0001
Number of comorbidity	1.21	1.06	−1.38	0.005	1.21	1.06	−1.38	0.005
CD8^+^CD28−CD27^+^ (tertile scores: 1,2,3)	1.42	1.11	−1.81	0.005	1.16	0.73	−1.86	0.53
CD8^+^CD28−CD27^+^ (low tertile)	1	—	—	—	1	—	—	—
CD8^+^CD28−CD27^+^ (mid tertile)	0.85	0.52	−1.39	0.512	0.81	0.27	−2.44	0.714
CD8^+^CD28−CD27^+^ (high tertile)	1.72	1.03	−2.87	0.037	2.56	0.96	−6.81	0.060

Abbreviations: CI, confidence interval; OR, odds ratio.

Multinomial logistic regression models were employed with sex, age and number of comorbidities as fixed variables in the base model, The results are shown for forward conditional stepwise selections of immune markers (tertiles of CD4/CD8 ratio, CD4^+^CD28^−^CD27^+^, CD8^+^CD28^−^CD27^+^ and CD8^+^CD28−) with *P*<0.05 for entry and 0.10 for retention), and identical results were obtained with backward selection procedures, to produce final prediction model of frailty.
