# Supplementary material for: Markers of T-cell senescence and physical frailty: insights from Singapore Longitudinal Ageing Studies
Source: NPJ Aging Mech Dis. 2015 Sep 28;1:15005–. doi: 10.1038/npjamd.2015.5 (PMC5514983; doi:10.1038/npjamd.2015.5)
Supplement: Supplementary Information [file npjamd20155-s1.doc]

**SUPPLEMENTARY INFORMATION**

**TABLES (S1, S2, S3) AND FIGURES (S1 and S2)**

**Table S1. Clinical and personal characteristics, and immune cell population subsets among robust, pre-frail and frail participants (N=421)**

|  | Numbers | (Percent) |  |  |  |
| --- | --- | --- | --- | --- | --- |
|  | Mean | ±SD | Median | Kurtosis | Skewness |
|  |  |  |  |  |  |
| Frailty score | 0.81 | ±0.98 | 1 | 1.23 | 1.14 |
| Robust | 202 | (48.0) |  |  |  |
| Pre-frail | 187 | (44.4) |  |  |  |
| Frail | 32 | (7.6) |  |  |  |
|  |  |  |  |  |  |
| Female sex | 218 | (51.8) |  |  |  |
| Age, mean ± SD | 66.5 | ±7.72 |  |  |  |
| Medical morbidities, mean ± SD | 2.04 | ±1.65 |  |  |  |
| Medical morbidities: 0-1 | 179 | (42.5) |  |  |  |
| 2-4 | 202 | (47.9) |  |  |  |
| >=5 | 39 | (9.3) |  |  |  |
|  |  |  |  |  |  |
| %CD4 | 71.64 | ±17.69 | 76.54 | -2.00 | 4.05 |
| %CD8 | 28.36 | ±17.69 | 23.46 | 2.00 | 4.05 |
| CD4/CD8 Ratio, mean ± SD | 3.62 | ±2.22 | 3.26 | 0.73 | 0.06 |
| CD4/CD8 Ratio <1, N (%) | 34 | (8.10) | NA | NA | NA |
| CD8+CD28+CD27- | 11.33 | ±8.66 | 9.00 | 1.65 | 3.59 |
| CD8+CD28+CD27+ | 46.03 | ±18.65 | 46.00 | -0.04 | -0.52 |
| CD8+CD28-CD27- | 27.40 | ±17.62 | 23.00 | 0.91 | 0.71 |
| CD8+CD28-CD27+ | 16.42 | ±12.78 | 13.00 | 1.63 | 2.97 |
| CD8+CD28- | 43.60 | ±22.60 | 40.00 | 0.83 | 0.96 |
| CD8+CD57+ | 37.14 | ±18.25 | 35.00 | 0.46 | -0.38 |
| CD4+CD28+CD27- | 13.89 | ±10.64 | 12 | 3.38 | 16.80 |
| CD4+CD28+CD27+ | 79.29 | ±13.67 | 82 | -2.45 | 9.85 |
| CD4+CD28-CD27- | 3.53 | ±5.10 | 2 | 4.28 | 30.71 |
| CD4+CD28-CD27+ | 3.16 | ±4.51 | 1 | 2.27 | 6.71 |
| CD4+CD27- | 17.42 | ±12.80 | 15 | 3.13 | 15.04 |
| CD4+CD57+ | 4.39 | ±4.66 | 3 | 4.71 | 39.92 |
|  |  |  |  |  |  |

Footnote: Medical comorbidities were defined by the number of chronic medical conditions form self-reports of having been diagnosed or treated by physician for a medical health problem. Figure for T cell subsets are percentage units ± SD

**Table S**2. Components of frailty in robust, pre-frail and frail participants.

|  | Robust | |  | Pre-frail | |  | Frail | |  |  |
| --- | --- | --- | --- | --- | --- | --- | --- | --- | --- | --- |
|  | N=202 | |  | N=187 | |  | N=32 | |  | P |
|  |  |  |  |  |  |  |  |  |  |  |
| Shrinking, N (%) | 0 | (0) |  | 20 | (10.7) |  | 8 | (25.0) |  |  |
| Slowness, N (%) | 0 | (0) |  | 55 | (29.4) |  | 30 | (93.8) |  |  |
| Weakness, N (%) | 0 | (0) |  | 73 | (39.0) |  | 30 | (93.8) |  |  |
| Exhaustion, N (%) | 0 | (0) |  | 57 | (30.5) |  | 20 | (62.5) |  |  |
| Inactivity, N (%) | 0 | (0) |  | 35 | (18.7) |  | 15 | (46.9) |  |  |
|  |  |  |  |  |  |  |  |  |  |  |
| Female, N (%) | 124 | (61.4) |  | 114 | (61.0) |  | 22 | (68.8) |  | 0.759 |
| Age, years, mean ±SD | 65.2 | ±6.7 |  | 66.5 | ±7.8 |  | 74.5 | ±8.6 |  | <0.0001 |
| Multi-morbidity, N (%) | 13 | (10.5) |  | 18 | (15.8) |  | 9 | (28.1) |  | 0.004 |
|  |  |  |  |  |  |  |  |  |  |  |

Footnote: Multimorbidity was defined as having more than 5 medical conditions. P values of statistical significance were derived from analysis of variance for continuous variables and chi-squared tests for categorical variables. *: p<0.01, Frail versus Prefrail; ^†^: p<0.01, Frail vs Robust (Bonferroni pairwise comparison)

**Table S**3. Co-morbid medical conditions among study participants (Robust, Pre-frail and Frail)

|  | Robust | |  | Pre-frail | |  | Frail | |  |  |
| --- | --- | --- | --- | --- | --- | --- | --- | --- | --- | --- |
| Medical conditions | N=202 | |  | N=187 | |  | N=32 | |  |  |
|  | N | % |  | N | % |  | N | % |  | p |
|  |  |  |  |  |  |  |  |  |  |  |
| Hypertension | 84 | 41.6 |  | 90 | 48.1 |  | 20 | 62.5 |  | 0.045 |
| Dyslipidemia | 99 | 49.0 |  | 95 | 50.8 |  | 16 | 50.0 |  | 0.92 |
| Diabetes | 25 | 12.4 |  | 34 | 18.2 |  | 10 | 31.3 |  | 0.014 |
| Stroke | 2 | 1.0 |  | 10 | 5.3 |  | 5 | 15.6 |  | <0.0001 |
| Heart disease | 12 | 5.9 |  | 7 | 3.7 |  | 3 | 9.4 |  | 0.341 |
| Cataract, glaucoma, eye diseases | 53 | 26.2 |  | 65 | 34.8 |  | 20 | 62.5 |  | <0.0001 |
| Cancer | 5 | 2.5 |  | 6 | 3.2 |  | 1 | 3.1 |  | 0.90 |
| Kidney failure | 0 | 0.0 |  | 3 | 1.6 |  | 1 | 3.1 |  | 0.034 |
| Asthma/COPD | 6 | 3.0 |  | 10 | 5.3 |  | 5 | 15.6 |  | 0.008 |
| Arthritis | 26 | 12.9 |  | 27 | 14.4 |  | 8 | 25.0 |  | 0.16 |
| Osteoporosis | 10 | 5.0 |  | 11 | 5.9 |  | 5 | 15.6 |  | 0.054 |
| Hip fracture | 1 | 0.5 |  | 0 | 0.0 |  | 1 | 3.1 |  | 0.054 |
| Dementia | 1 | 0.5 |  | 1 | 0.5 |  | 1 | 3.1 |  | 0.240 |
| Parkinson’s disease | 1 | 0.5 |  | 2 | 1.1 |  | 1 | 3.1 |  | 0.49 |
| Gastro-intestinal problems | 10 | 5.0 |  | 15 | 8.0 |  | 4 | 15.6 |  | 0.032 |
| Thyroid disorders | 8 | 4.0 |  | 9 | 4.8 |  | 2 | 6.3 |  | 0.80 |
| Depression | 3 | 1.5 |  | 6 | 3.2 |  | 1 | 3.1 |  | 0.288 |
| Others | 37 | 18.3 |  | 46 | 24.6 |  | 8 | 25.0 |  | 0.144 |
|  |  |  |  |  |  |  |  |  |  |  |


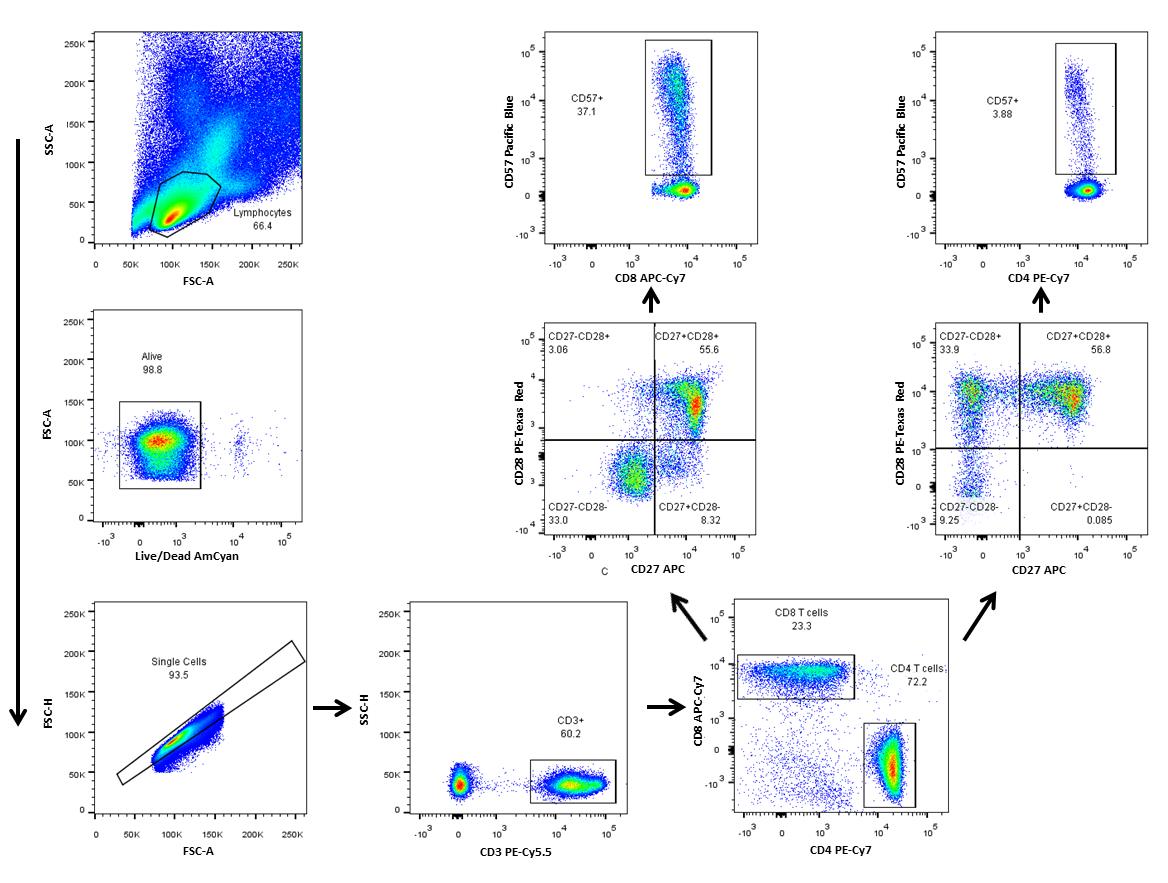


**Figure S1. Gating strategy for T cell populations.** After acquisition by flow cytometry, samples were analyzed for the expression of markers of interest using multiple steps gating strategy. We first eliminated non-lymphocytes, dead cells, then include only single cells and CD3+ cells (gates 1, 2, 3, and 4 respectively). We then separated the CD3+ population into CD4+ and CD8+ cells (step 5) and measured the expression of CD27/CD28 and CD57 on each population. Percentage reported are always for the parent population.


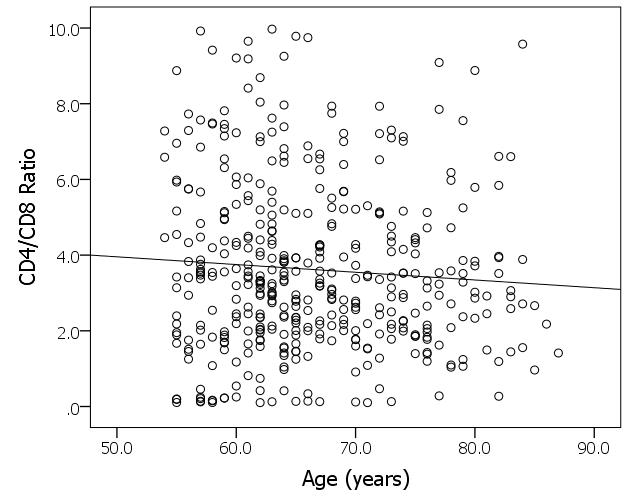

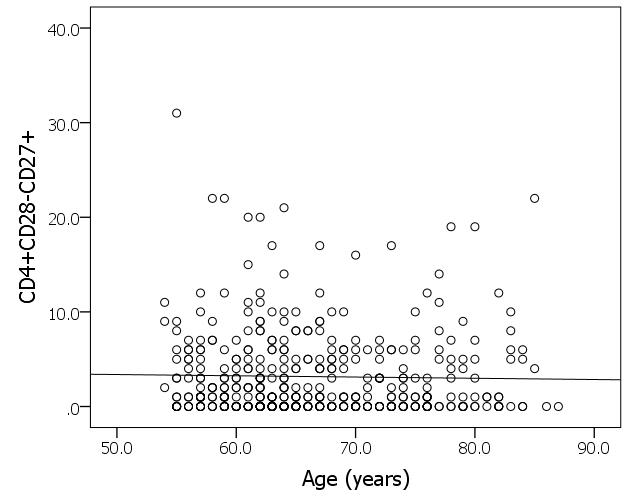


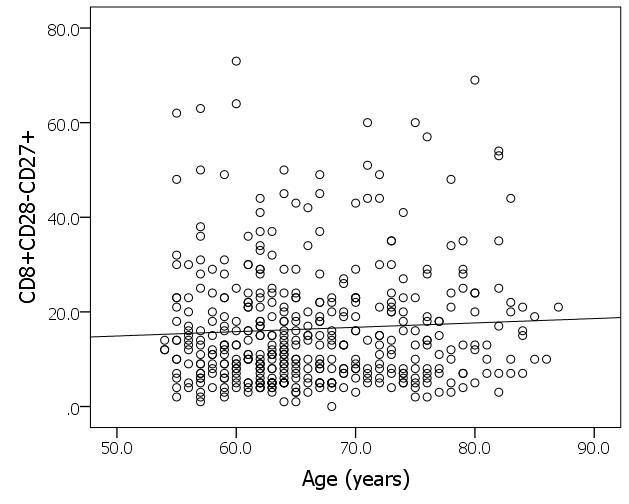

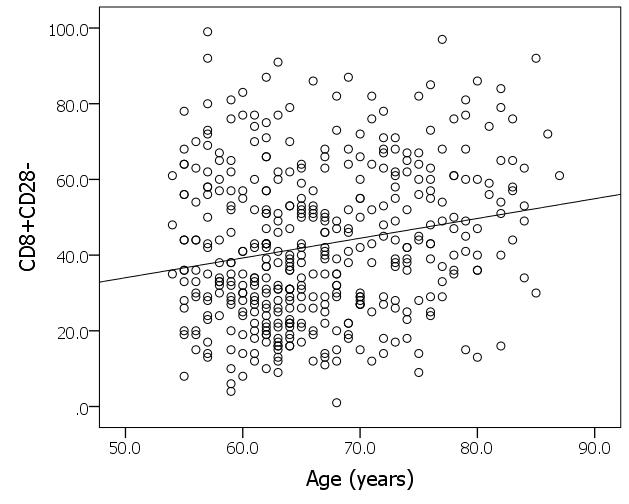


**Figure S2**  Relationships with age of T cell subsets CD8+CD28-CD27+, CD4+CD28-CD27+ T cells, CD8+CD28-, and CD4/CD8 ratio.
